# Identification of IGF2BP3 and CENPA as key regulators of immunophenotypes in renal clear cell carcinoma

**DOI:** 10.3389/fgene.2025.1749780

**Published:** 2026-01-12

**Authors:** Tianjie Zhu, Liying He, Shuai Li, Jingyuan Zhao

**Affiliations:** 1 Central Hospital of Dalian University of Technology, Dalian, China; 2 Chongqing Institute for Food and Drug Control, Chongqing, China; 3 Department of Pharmacy, The First Affiliated Hospital of Dalian Medical University, Dalian, China

**Keywords:** CenpA, IGF2BP3, immunophenotype, KIRC, prognostic biomarker

## Abstract

**Introduction:**

Kidney renal clear cell carcinoma (KIRC) is the most common subtype of Renal cell carcinoma (RCC), with a high degree of immune infiltration. This study aimed to identify m6A-related biomarkers and downstream effectors in KIRC that may affect tumor immunity and to provide prognosis biomarkers of KIRC.

**Methods:**

In this study, the mRNA expression profiles and corresponding clinical data of KIRC patients were downloaded from The Cancer Genome Atlas (TCGA) to screen out transcription factors and m6A-related genes that were upregulated and unfavorable to the prognosis of KIRC. The multigene signature was constructed using LASSO analysis to selected two transcription factors and a m6A-associated gene, and TCGA cohort was constructed to stratify patients into two risk groups.

**Results:**

Functional analysis showed that immune-related pathways were enriched and that immune status was different between the two risk groups, with Insulin-like growth factor 2 mRNA-binding protein 3 (IGF2BP3) and centromere protein A (CENPA) genes highly correlated with immune cell infiltration.

**Discussion:**

We found that silencing CENPA significantly increased reactive oxygen species production and mitochondrial membrane potential abnormalities leading to inhibition of cell viability and proliferation and cell death, suggesting that CENPA is closely associated with the development of KIRC. In conclusion, IGF2BP3 and its downstream CENPA signature can be used for prognostic prediction of KIRC.

## Introduction

Renal cell carcinoma (RCC) originates from the renal epithelium and its incidence is increasing every year, accounting for more than 90% of all kidney cancers. Kidney renal clear cell carcinoma (KIRC) is the most common subtype of RCC, accounting for 60%–85% of all RCC, and has a relatively high degree of immune infiltration (Senbabaoglu et al., 2016). In recent years, the emergence of drugs that target Programmed cell death protein 1 (PD-1) and Programmed Cell Death Ligand 1 (PD-L1), the treatment of kidney cancer has marked a shift from the era of targeted therapy to the era of immunotherapy. Immune checkpoint therapy (ICT) and combination regimens offer lasting clinical benefits for patients with advanced KIRC. However, KIRC is highly heterogeneous, with the composition of tumor cells and other cells in the tumor microenvironment at the same histological stage varying considerably from patient to patient (Braun et al., 2021). As a result, many patients will exhibit primary or adaptive resistance to ICT. Therefore, it is crucial to dive deep into the underlying molecular mechanisms of KIRC and identify immunotherapy-related biomarkers for implementing precise treatment for KIRC patients.

RNA modification is a key process in epigenetics that regulates post-transcriptional gene expression. Among them, N6-methyladenosine (m6A) is the most abundant form of mRNA modification in eukaryotes, which is involved in regulating the stability, splicing, degradation, translation and other processes of mRNAs (Wang et al., 2022; Ma et al., 2019). The m6A methylation modification is mainly mediated through the methyltransferases writer METTL3 and METTL14, and the demethylases eraser FTO and ALKBH5. At the same time, m6A modification is also dependent on the m6A-binding proteins reader, which recognizes and identifies m6A-modified mRNAs and thus guides the biological function of mRNAs. The m6A binding proteins consist of a set of proteins containing the YT521-B homology (YTH) domain (YTHDF1, YTHDF2, YTHDF3, YTHDC1, YTHDC2), insulin-like growth factor 2 (IGF-2) mRNA binding proteins (IGF2BP1, IGF2BP2, IGF2BP3), the heterogeneous nuclear ribonucleoproteins hnRNPA2B1 and hnRNPC, and other components (Zhu S. et al., 2020). Among them, IGF2BP3 has high expression levels and is associated with poor survival rates in various cancer types, and it has been identified as a potential oncogene for multiple types of cancer (Li et al., 2019). IGF2BP3 may help stabilize the mRNAs stability of target genes via its m6A modification site (Hu et al., 2022; Sun et al., 2022), thereby promoting cancer development and progression.

We define “m6A-related biomarkers” as molecules whose biological functions, expression regulation, or oncogenic mechanisms involve direct or critically indirect interactions with the m6A RNA methylation pathway. IGF2BP3 is widely recognized as a canonical m6A “reader” protein and is therefore an integral component of the m6A regulatory machinery. The association between IGF2BP3 and m6A regulation is manifested at three levels: molecular mechanism (direct recognition and dependence on m6A modification), functional role (modulating RNA metabolism and fate via m6A), and disease relevance (serving as a central m6A effector in cancer). The IGF2BP3 protein specifically recognizes and binds RNA sequences containing m6A modifications through its KH domains. Research by Huang et al. provided the first systematic demonstration that IGF2BP family proteins, including IGF2BP3, directly recognize m6A modifications and significantly enhance the stability and translation efficiency of target mRNAs. Their study confirmed direct binding of IGF2BP3 to m6A sites using photoactivatable ribonucleoside-enhanced crosslinking and immunoprecipitation sequencing (Huang et al., 2020). Upon binding to m6A modifications, IGF2BP3 promotes cancer by recruiting specific adaptor proteins to protect target mRNAs from degradation by exonuclease complexes. This mechanism is considered central to its oncogenic function. Supporting this view, studies have demonstrated that IGF2BP3 maintains the stability of target mRNAs by inhibiting their deadenylation process—a function strictly dependent on its interaction with m6A modifications. Knockout of the m6A methyltransferase METTL3 abolishes this stabilizing effect of IGF2BP3 (Müller et al., 2018). In various cancers, IGF2BP3 recognizes m6A modifications on a set of key oncogenic transcripts (such as MYC, SOX2, EGFR, etc.), forming an “m6A–IGF2BP3–oncogene” axis that drives tumorigenesis and progression, underscoring its role as a key effector protein linking m6A modification to cancer phenotypes (Zhan et al., 2023; Zhang et al., 2022; Liu et al., 2023). Therefore, in KIRC and various other cancers, IGF2BP3 utilizes its m6A-reading function to stabilize a cohort of oncogenic transcripts, thereby driving tumor proliferation, invasion, and metastasis.

The m6A modifications are not only closely related to tumorigenesis and development but also play an important role in immune regulation (Su et al., 2020; Lou et al., 2021). However, the role of m6A in KIRC immune evasion remains uncharacterized. Identifying optimal m6A-related biomarkers and their downstream effector is vital to maximizing the treatment efficacy of immunotherapy.

In this study, to seek potential m6A-related biomarkers and their downstream effector for KIRC, we processed a series of analyses based on high‐throughput sequencing data obtained from public tumor databases. As a result, we demonstrated that m6A-related biomarkers (IGF2BP3) and transcription factor (CENPA) are possible biomarkers of KIRC, which are all related to the prognosis of patients with KIRC. Based on these findings, further investigation into their functional roles may pave the way for the development of novel diagnostic biomarkers and therapeutic strategies for KIRC.

## Materials and methods

### Data collection

The gene expression profiles of KIRC were downloaded from The Cancer Genome Atlas (TCGA) database (https://portal.gdc.cancer.gov/) and GEO dataset GSE40435. Updated clinical data related to these samples, such as age, gender, race, overall survival time and vital status, were also downloaded from the TCGA. A comprehensive list of transcription factors (TF) was acquired from Cistrome Cancer (http://cistrome.org/CistromeCancer/). We obtained m6A-related genes from the literature (Additional file Supplementary Table S1). Subsequently, the gene expression was screened for subsequent analysis by the R software (version 4.1.2).

### Identification of differentially expressed genes (DEGs)

We screened DEGs between tumor samples from TCGA-KIRC and normal tissues using the limma package in the R software. An adjusted p-value <0.05 and |log2 (FC)| value > 1 was considered significant (Additional file Supplementary Table S2). We took the intersection of DEGs and m6A genes to obtain differentially expressed m6A-related genes (DEm6As). We investigated the intersection between DEGs and TFs to facilitate further screening for differentially expressed TF genes (DETF) involved in the development and progression of KIRC.

### Construction of the prognostic risk gene signature

We analyzed the overall survival (OS) and disease-free survival (DFS) of m6As by Gene Set Cancer Analysis (GSCA) (http://bioinfo.life.hust.edu.cn/GSCA/#/) which is an integrated platform for genomic, pharmacogenomic, and immunogenomic gene set cancer analysis (Liu et al., 2018). Univariate Cox analysis of OS was performed to screen DETFs-related and DEm6As-related genes with prognostic values. The TCGA-KIRC was used to clarify the potential prognostic significance of these OS-related genes in KIRC patients. To avoid overfitting the prognostic risk signature, we used the least absolute shrinkage and selection operator (LASSO)-based Cox regression on the training dataset to identify the most significant features within the OS-related genes. Penalty parameter (λ) for the model was determined by tenfold cross-validation following the minimum criteria (The value of λ corresponding to the lowest partial likelihood deviance). The risk scores of the patients were calculated according to the expression level of each gene and its corresponding regression coefficients. The patients were stratified into high and low-risk groups based on the median value of the risk score. The “survival ROC” R package was used to conduct time-dependent receiver operating characteristic (ROC) curve analyses to evaluate the predictive power of the gene signature. Kaplan-Meier survival curves showed the difference in OS between the high- and low-risk groups, which were stratified based on the gene signature.

### DEGs analysis and enrichment analysis of KIRC between the high-risk and low-risk groups

Expression profiles were compared between high- and low-risk groups to identify DEGs using the R software “limma” Package. Genes with |log2FC|>1 and p-value<0.05 were considered as DEGs. Gene Ontology (GO) and Kyoto Encyclopedia of Genes and Genomes (KEGG) analyses based on the DEGs were conducted by clusterProfiler package. KEGG pathway enrichment analysis was performed using the KEGG database (Kyoto Encyclopedia of Genes and Genomes, https://www.kegg.jp/) (Kanehisa et al., 2025).

### Exploration of tumor immune infiltration

To explore the degree of immune cell infiltration, the ESTIMATE algorithm (https://sourceforge.net/projects/estimateproject/) was applied to calculate the estimate scores, immune scores, and stromal scores. Furthermore, the OS cases were assigned to high- and low immune score groups based on the median value of immune scores, to identify a possible association of immune score with overall survival. CIBERSORT was used to calculate the infiltration of 22 immune cell types in the KIRC, and the difference between high-risk and low-risk groups was analyzed.

### Protein-protein interaction (PPI) network analysis

STRING online database (http://string-db.org) and the Cytoscape software were used to construct the PPI network. The relationship between the prognostic risk genes and target genes was exhibited in the PPI network.

### Gene set enrichment analyses (GSEA)

To further explore the potential molecular mechanisms, GSEA was performed using GSEA 4.02 (http://www.broad.mit.edu/gsea/). Pathway with the nominal p < 0.05 and FDR <0.25 were considered statistically significant.

### Validation of the target genes based on TCGA data

UALCAN (http://ualcan.path.uab.edu) was used to verify the survival and expression patterns between tumor and normal groups in our results. We analyzed the relationship between mRNA expression of the risk gene with clinicopathological parameters of KIRC, including patients’ individual cancer stages and tumor metastasis status. The methylation degree of DNA of the gene was analyzed by GSCA. Subsequently, the pearson correlation was calculated between the target gene methylation and mRNA expression.

### Cell culture and siRNA transfection

The KIRC 769P cells (Cat# CL-0009, RRID: CVCL_1050) purchased from Procell (China) were cultured in DMEM (Gibco, USA) supplemented with 10% FBS (Gibco, USA), 1% penicillin, and 1% streptomycin, and cells were maintained in a 37 °C incubator with 5% CO_2_.

siRNAs targeting CENPA, IGF2BP3 or a negative control (NC) were designed and synthesized by GenePharma (China). In brief, cells (2 × 10^6^) were cultured in 6-well plates. The reagent containing siRNA (20 µM) and lipo 3000 (Thermo Fisher, USA), which was previously incubated for 15 mins, were mixed and then added to cells. After 48 h of incubation, the levels of target proteins were examined by Western blot analysis.

### Western blotting

The CENPA (Cat# 26754-1-AP), IGF2BP3 (Cat# 81805-1-RR) and GAPDH (#10494-1-AP) antibodies were purchased from Proteintech. Proteins were electrophoresed in SDS-PAGE gel and transferred onto PVDF membranes. The membranes were blocked with 5% skim milk in TBS/0.1% Tween 20 containing for 0.5 h and then incubated overnight with the primary antibodies at 4 °C. The membrane was further incubated with HRP-conjugated secondary antibodies for 1 h at room temperature. Protein bands were visualized with ECL substrates.

### Cellular viability assay

To assess the effect of CENPA on cell viability, 769P cells were stained with calcein-AM/PI for 30 min. Finally, the labeled cells were rinsed twice with PBS and imaged (excitation wavelengths: 488 nm and 530 nm for calcein-AM and PI, respectively).

### Colony formation

500 769P cells were inoculated into each well of a 6-well plate and maintained in a medium containing 10% FBS for 10 days. Colonies were fixed with 4% paraformaldehyde, stained with 0.1% crystal violet solution, and counted under an inverted microscope. Three replicate wells were set up for each group of experiments.

### Mitochondrial activity assay

Mitochondrial activity was measured with the following two dyes: JC1 (MCE, USA). For JC1 staining, live cells were stained with JC1 (10 μM) at 37 °C for 30 min, and the fluorescence was observed and imaged using fluorescence microscopy.

### Intracellular reactive oxygen species assay

Intracellular reactive oxygen species (ROS) production was detected using DCFH-DA (MCE). Briefly, after 769P cells were stained with 10 μM DCFH-DA for 20 min at 37 °C. Hoechst 33,342 was used to stain cells for 10 min, and images were acquired with a fluorescence microscope.

### Statistical analyses

Results are recorded as means ± standard error of the mean for at least three independent experiments and analyzed by R software (Version 4.1.2) or GraphPad Prism 8 software. The Wilcoxon rank-sum test and the Kruskal-Wallis test were used for the comparison of continuous variables. Pearson analysis was used for the correlation analyses. Data were considered statistically significant as follows: *P < 0.05, **P < 0.01, ***P < 0.001 and ****P < 0.0001.

## Result

### Identification of DETFs and DEm6As in KIRC

**FIGURE 1:**
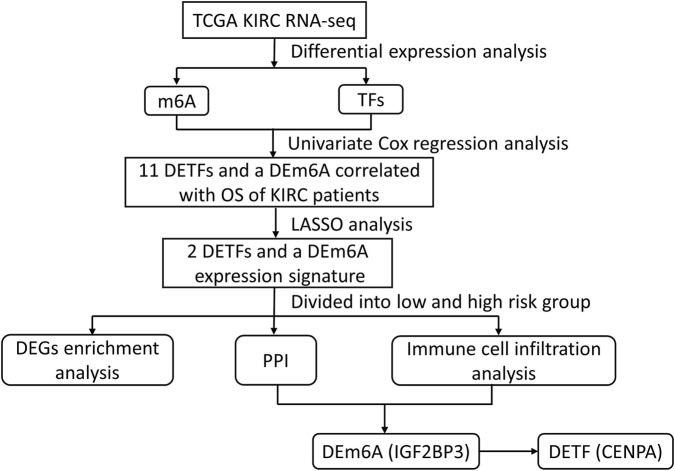
Flow chart of data collection and analysis.

**FIGURE 2:**
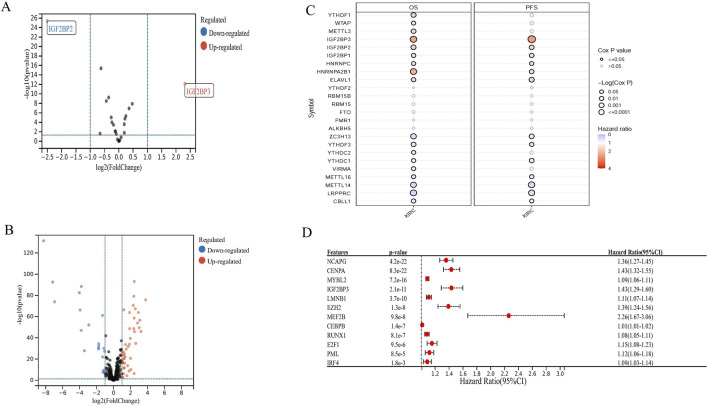
Identification of DEGs between tumor samples and normal tissues based on the TCGA database. **(A)** A volcano plot of DEm6As. **(B)** A volcano plot of DETFs. **(C)** Prognostic analyses of OS and PFS of all m6As. **(D)** Forest plot showing the result of the univariate Cox analyses for 11 DETFs with a DEm6A that was incorporated into the signatures.

The flow chart of the study is shown in Figure 1. We obtained DEGs and 2 DEm6As in the TCGA dataset, IGF2BP3 was up-regulated and IGF2BP2 down-regulated (Figure 2A). To validate the data in TCGA, we also examined the expression of these genes in the GEO dataset GSE40435 in KIRC, which further supports the upregulation of IGF2BP3 in tumors (Supplementary Figure S1). The intersection of the TFs and DEGs in KIRC identified 65 DETFs, of which 44 genes were upregulated and 21 downregulated (Figure 2B).

We analyzed the impact of m6A-related genes on survival in KIRC. Notably, IGF2BP3 was found to significantly promote cancer progression and showed strong clinical relevance in both overall survival (OS) and progression-free survival (PFS) cohorts (Figure 2C). In addition, 11 differentially expressed transcription factors (DETFs) were identified as being significantly associated with OS in KIRC, all exhibiting hazard ratios (HR) greater than 1, suggesting their role in promoting tumor growth (Figure 2D).

### Development of OS-related risk signature

**FIGURE 3:**
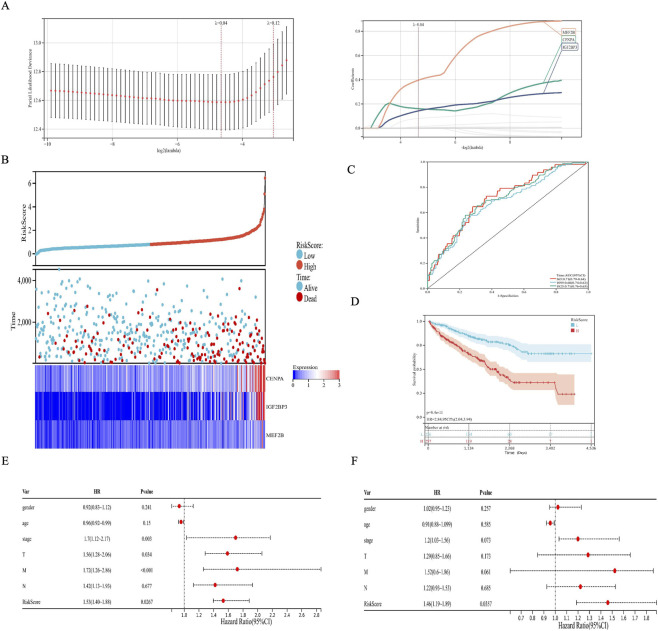
Construction of the risk prognostic classifier. **(A)** LASSO coefficient profiles of the risk genes. **(B)** The risk scores and heatmap of expression profiles in high-risk and low-risk groups. **(C)** AUC of time-dependent ROC curves of the 3-risk genes prognostic signature. **(D)** OS of KIRC patients in high-risk and low-risk groups. **(E)** Univariate and **(F)** multivariate independent prognostic analysis in patients with KIRC.

Eleven DETFs and a DEm6A further performed LASSO regression. LASSO analysis identified three genes (IGF2BP3, CENPA and MEF2B) which were included in the classifier (Figure 3A). We constructed a multi-gene risk prediction model incorporating IGF2BP3, CENPA, and MEF2B to evaluate the prognostic predictive power of these three key genes in clear cell renal cell carcinoma (KIRC). The independent risk weight of each gene was determined by Cox proportional hazards regression analysis, and a comprehensive risk score was calculated for each patient accordingly. Using the median of the risk score as the cut-off point, patients were stratified into high-risk and low-risk groups (Figure 3B). The analysis results showed that the number of deaths was significantly higher in the high-risk group compared to the low-risk group, and the high-risk group had higher levels of CENPA, IGF2BP3 and MEF2B expression (Figure 3B). The areas under the curve (AUC) for the risk model in predicting 1, 3 and 5-year survival were 0.71, 0.68, and 0.71, respectively (Figure 3C). Moreover, a high risk score was significantly associated with poor outcomes (Figure 3D), revealing this score as a good predictive tool. Univariate (Figure 3E) and multivariate (Figure 3F) independent prognostic analysis showed that the risk score was the only independent predictor for KIRC, indicating the great performance of this index.

Existing studies have directly elucidated the role of IGF2BP3 in clear cell renal cell carcinoma (KIRC). For instance, functional research by Li et al. (2021) demonstrated that IGF2BP3 promotes the proliferation, migration, and invasion of KIRC cells by stabilizing UHRF1 mRNA. Additionally, Xie et al. (2021) reported that IGF2BP3-mediated LncRNA CDKN2B-AS1 epigenetically activates NUF2 transcription, thereby driving malignant progression in KIRC. These findings collectively provide direct experimental evidence supporting the pro-oncogenic function of IGF2BP3 in KIRC. CENPA is a centromere-specific histone H3 variant and serves as the core component of the chromosomal passenger complex, playing a critical role in accurate chromosome segregation. Its overexpression leads to chromosomal instability and aneuploidy, which are classic hallmarks of cancer. Recent studies have confirmed that CENPA is highly expressed in clear cell renal cell carcinoma (KIRC) and is associated with poor prognosis as a biomarker. CENPA promotes cancer progression and metastasis by accelerating the cell cycle and activating the Wnt/β-catenin signaling pathway (Wang et al., 2021). MEF2B, a member of the myocyte enhancer factor 2 family, is involved in regulating cell differentiation, proliferation, and apoptosis. It has been extensively studied in lymphoma and has been shown to function as an oncogene during the development of B-cell lymphoma (Yu et al., 2024; Brescia et al., 2018). Although direct functional studies of MEF2B in KIRC are limited, Huang et al. found that high expression of MEF2B is significantly associated with poor patient prognosis and verified that it promotes the proliferation, migration, and invasion of KIRC cells *in vitro* (Li et al., 2024). Based on the above findings, we propose a mechanistic hypothesis: MEF2B may participate in the regulation of the IGF2BP3/CENPA axis and potentially promote KIRC progression by activating specific transcriptional programs. Specifically, MEF2B might function as an upstream transcriptional regulator that directly activates the transcription of IGF2BP3 or CENPA, thereby amplifying the function of this network at the transcriptional level. Alternatively, MEF2B may act in parallel with IGF2BP3 (via m6A modification) and CENPA (via regulation of chromosomal stability), collectively converging on downstream pathways that drive cell cycle dysregulation and genomic instability.

### The immune score is associated with overall survival in high-risk groups

**FIGURE 4:**
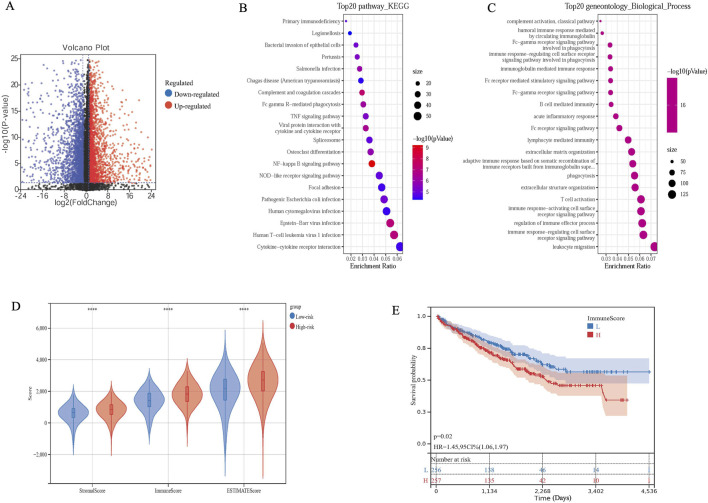
DEGs and Immune characteristics between the high-risk and the low-risk groups. **(A)** Volcano plot of the DEGs. **(B)** Significantly enriched KEGG pathways of the DEGs. **(C)** Significantly enriched Gene Ontology terms of the DEGs. **(D)** Immune characteristics in terms of risk core. **(E)** OS analysis of KIRC patients with different ImmuneScore.

Further differential genetic analysis was performed between high- and low-risk samples. A total of 4,714 DEGs were obtained, containing 2220 up-regulated genes and 2,494 down-regulated genes (Figure 4A). To elucidate the biological functions and pathways, the DEGs between the high-risk and low-risk groups were used to perform GO enrichment and KEGG pathway analyses. DEGs were enriched in KEGG pathway including cytokine receptor and cytokine-cytokine receptor intersection (Figure 4B). The DEGs were also obviously enriched in many immune-related biological processes, including immune response-regulating cell surface receptor signaling pathway and leukocyte migration (Figure 4C).

The m6A modification serves as an epigenetic regulator with significant and varied biological functions, particularly in modulating the tumor immune microenvironment (Mu et al., 2024; Han et al., 2024; Luo et al., 2022; Sheng et al., 2023). Given that IGF2BP3 is known to influence this microenvironment (Liu et al., 2023), we are analyzing prognostic models’ impact on KIRC immunity to assess the roles of IGF2BP3, CENPA, and MEF2B axis as a whole factor in tumor immunity. We determined ImmuneScore, StromalScore and ESTIMATEScore of the high-risk and low-risk groups by applying the ESTIMATE algorithm. A higher ImmuneScore and StromalScore were represented in the high-risk group (Figure 4D). Subsequently, the cases were assigned to the high and low immune score groups respectively, according to the median value of immune scores. We utilized the Kaplan-Meier survival analysis, the results revealed that a low immune score was significantly associated with improved outcomes (Figure 4E). These findings clarify that the immune component plays a key role in the progression of the high-risk and low-risk groups.

Further comparative analysis revealed that the previously defined high-risk group, despite generally exhibiting higher immune scores, demonstrated lower survival rates. This seemingly paradoxical phenomenon likely reflects the complexity of the tumor microenvironment (TME): a higher immune score may not simply indicate enhanced anti-tumor immunity but is more likely associated with the enrichment of immunosuppressive cell populations (such as regulatory T cells [Tregs] and M2-type tumor-associated macrophages [TAMs]) or elevated expression of co-inhibitory molecules (PD-1/PD-L1). Consequently, this fosters an immunosuppressive and dysfunctional microenvironment. Such an inhibitory immune state ultimately impairs effective anti-tumor immune responses, leading to poorer patient prognosis.

### Tumor infiltrating immune cells and PPI network analysis

**FIGURE 5:**
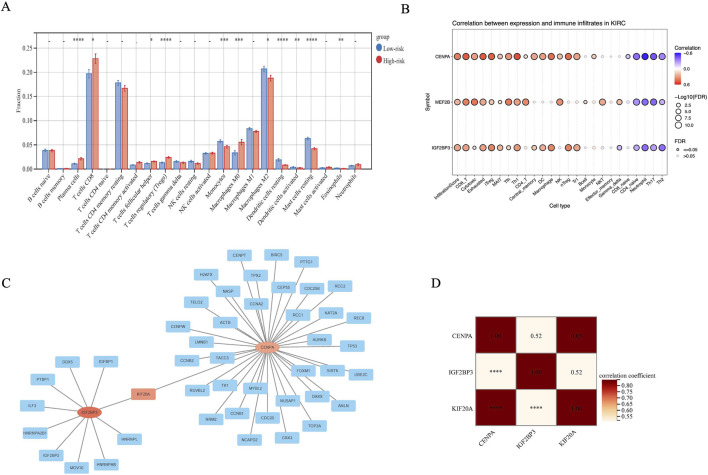
Tumor-immune micro environment analysis of the high-risk and low-risk groups. **(A)** Bar graph comparing the proportions of immune cells between low- and high-risk OS samples. The red color represents the high-risk group while the blue color represents the low-risk group. **(B)** Correlation heatmap of tumor-infiltrating immune cells and three risk genes. **(C)** The PPI network of the risk genes was constructed using Cytoscape. **(D)** The correlation among three risk genes.

Next, we investigated the levels of immune cell infiltration in high-risk and low-risk groups, we found that the proportions of T cells regulatory (Tregs) were significantly higher in the high-risk group compared with the low-risk group (Figure 5A). Next we performed Spearman correlation analyses to explore the relationships between the genes and tumor-infiltrating immune cells. The results showed that the expression levels of IGF2BP3 and CENPA were positively correlated with the levels of immune exhausted and immunosuppressive nTreg and iTreg cells (Figure 5B), demonstrating the inhibitory role of IGF2BP3 and CENPA in tumor immunity, and that immune exhaustion may lead to poorer prognosis for patients.

IGF2BP3, as an m6A reader, plays a dual role in tumor immunity by regulating the stability of specific mRNAs. Studies have shown that in colorectal cancer, IGF2BP3 modulates the expression of T cell chemokines such as CCL5 and CXCL9/10, thereby influencing T cell recruitment to the tumor site (Yang et al., 2020). On the other hand, CENPA, as a core centromeric protein, primarily affects tumor immunity indirectly by inducing chromosomal instability. Research suggests that this instability can activate the cGAS–STING pathway, leading to type I interferon production and subsequent recruitment of CD8^+^ T cells (Bakhoum et al., 2018). Therefore, the increased infiltration of CD8^+^ T cells observed in the high-risk group may arise from CENPA-driven chromosomal instability activating the cGAS-STING pathway, coupled with the potential regulation of T-cell chemokines by IGF2BP3. However, these infiltrating T cells are likely rendered functionally exhausted due to the concurrent upregulation of immune checkpoint molecules such as PD-L1, which is mediated by IGF2BP3, thereby explaining their association with poor prognosis.

To better understand the potential biological function of the DEGs, we performed a PPI analysis, the network (Figure 5C) identified in the DEGs indicated that IGF2BP3 and CENPA may work through KIF20A which is involved in key cell functions including includes the intracellular movement of organelles and vesicles, spindle formation and cell division (Echard et al., 1998). KIF20A expression level and activity participate in the regulation of intracellular transport and cell division, which play an important role in carcinogenesis and development (Hirokawa et al., 1998).

Spearman correlation analysis showed the relationship between IGF2BP3, CENPA and KIF20A, and the results showed a significant correlation between these three genes (Figure 5D). Indeed, IGF2BP3 provides the material basis for aberrant cell proliferation and division, while CENPA, through its dysregulated expression, directly disrupts centromere function and the accuracy of chromosome segregation. KIF20A, as a kinesin motor protein, operates at the executive level to facilitate spindle assembly and cytokinesis completion. Although direct regulatory relationships among these three factors have not yet been elucidated in clear cell renal cell carcinoma (KIRC), independent studies have confirmed that each is a key driver of KIRC progression by compromising mitotic fidelity and dysregulating cell cycle progression (Li et al., 2021; Echard et al., 1998; Hirokawa et al., 1998; Wang et al., 2021; Zhu et al., 2021). We propose that they may constitute a functionally synergistic oncogenic module: IGF2BP3 lays the material foundation for abnormal mitosis through epitranscriptomic regulation, while CENPA and KIF20A execute the process at the levels of chromosome segregation and spindle function, respectively, ultimately leading to genomic instability and malignant tumor evolution.

### Gene sets enriched in CENPA expression phenotype

**FIGURE 6:**
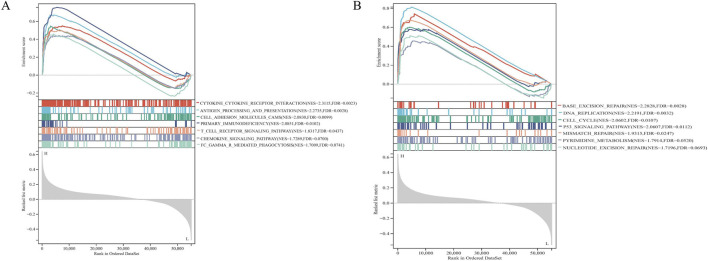
CENPA gene set enrichment plots of **(A)** Immune-related biological process. **(B)** Sustaining proliferative signaling pathway.

To characterize the potential function of the IGF2BP3 gene, we performed an analysis of the impact of RNA sequencing data on the pathways influenced by IGF2BP3, the results indicate that IGF2BP3 plays a regulatory role in proliferation, cell cycle, apoptosis, and DNA damage (Supplementary Figure S2). To characterize the potential function of the CENPA gene, GSEA was performed on the differential genes. GSEA identified cytokine-cytokine receptor intersection (NES = 2.3155, FDR = 0.0023), antigen processing and presentation (NES = 2.2735, FDR = 0.0028), cell adhesion molecules cams (NES = 2.0830, FDR = 0.0099), primary immunodeficiency (NES = 2.0851, FDR = 0.0102), T cell receptor signaling pathway (NES = 1.8317, FDR = 0.0437), chemokine signaling pathway (NES = 1.7289, FDR = 0.0700), Fc gamma R mediated phagocytosis (NES = 1.7009, FDR = 0.0741) in immune system of CENPA (Figure 6A). GSEA also identified base excision repair (NES = 2.2828, FDR = 0.0028), DNA replication (NES = 2.2191, FDR = 0.0032), cell cycle (NES = 2.0602, FDR = 0.0107), p53 signaling pathway (NES = 2.0607, FDR = 0.0112), mismatch repair (NES = 1.9315, FDR = 0.0247), pyrimidine metabolism (NES = 1.7196, FDR = 0.0693), nucleotide excision repair (NES = 1.7914, FDR = 0.0520) in proliferation of CENPA (Figure 6B). We also added further analysis using RNA seq data, indicating the role of CENPA in regulating apoptosis and the cell cycle. (Supplementary Figure S3). The results show that CENPA could participate in immune and proliferation, which is consistent with our above results.

### Validation of gene expression and DNA methylation levels

**FIGURE 7:**
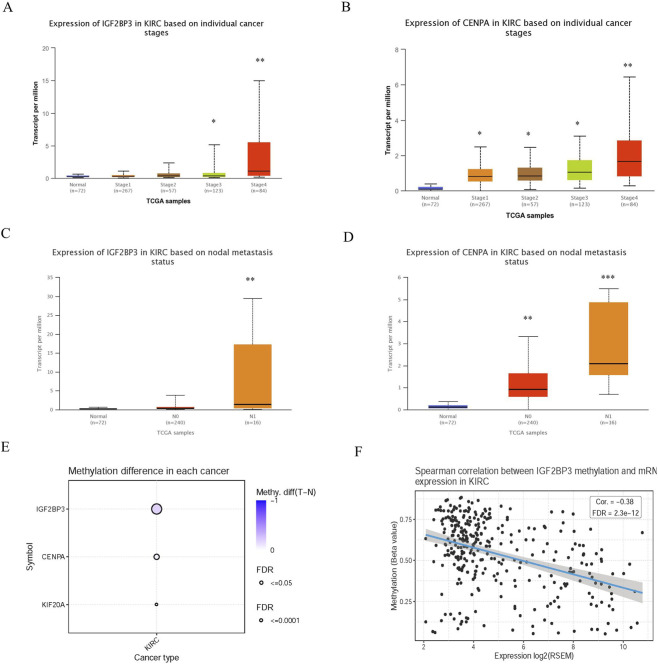
Expression levels and Methylation status of the two mRNAs. **(A)** Expression of IGF2BP3 in KIRC based on individual cancer stages. **(B)** Expression of CENPA in KIRC based on individual cancer stages. **(C)** Expression of IGF2BP3 based on nodal metastasis status. **(D)** Expression of CENPA based on nodal metastasis status. **(E)** Methylation difference of genes in KIRC. **(F)** Spearman correlation between IGF2BP3 methylation and mRNA expression in KIRC.

The expressions of genes were remarkably correlated with patients’ individual cancer stages, and patients who were in more advanced cancer stages tended to express higher mRNA expression (Figures 7A–D). Similarly, mRNA expressions of the two risk genes were significantly related to tumor metastasis, the highest mRNA expressions of the risk genes were found in the tumor metastasis group.

As was shown in Figure 7E, the DNA methylation degree of IGF2BP3 is much higher than that of CENPA in KIRC cancer. It was also found that IGF2BP3 methylation was negatively correlated with mRNA expression in KIRC (Figure 7F).

### Inhibition of CENPA expression leads to growth inhibition and death of KIRC cells

**FIGURE 8:**
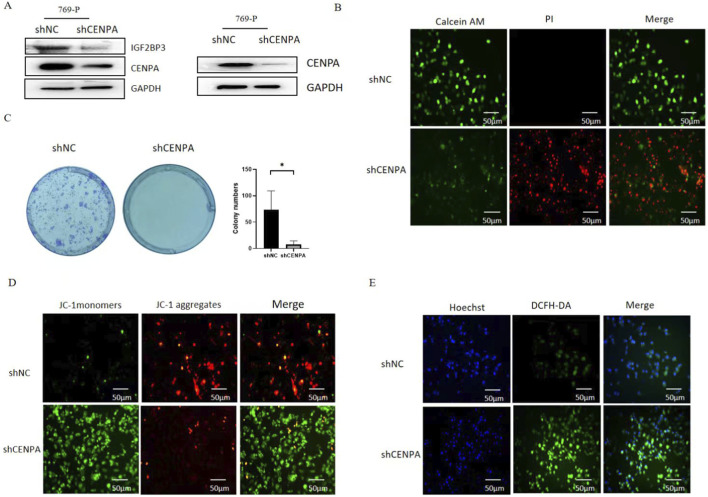
Inhibition of CENPA expression leads to growth inhibition and death of KIRC cells. **(A)** The expression of IGF2BP3 and CENPA were detected by Western blot. **(B)** Calcein-AM/PI assay was used to explore the effect of CENPA expression on cell viability. **(C)** Representative images and quantification of colony formation assay, *p < 0.05. **(D)** Mitochondrial membrane potential was analyzed by JC1. **(E)** Reactive oxygen species assay.

Given that we found CENPA to be associated with cell cycle and apoptosis by GSEA analysis, to verify the effect of CENPA on KIRC cell cells, siRNA targeting CENPA was transfected. The silencing efficiency was verified by Western blotting (Figure 8A). We used calcein-AM/PI to assess cell viability and showed that downregulation of CENPA significantly reduced the survival of 769P cells (Figure 8B). Colony formation assay revealed a significant difference in colony formation of cells silencing CENPA expression compared to the control group (Figure 8C), suggesting that CENPA affects tumor growth. To further confirm the effect of CENPA on cell growth and death, we analyzed the mitochondrial membrane potential using JC1. The results showed that CENPA had an effect on the mitochondrial membrane potential of 769P cells (Figure 8D), and the reactive oxygen species assay revealed that silencing CENPA significantly increased the reactive oxygen species level of the cells (Figure 8E). These results suggest that CENPA affects the growth and death of KIRC cells. Mitochondria, as organelles with various biological functions within cells, play a key role in regulating metabolism and activating immune cells (Mills et al., 2017). Reactive oxygen species (ROS) can not only quickly eliminate tumors but also induce immunogenic cell death (ICD), triggering the body’s anti-tumor immune response (He et al., 2023). The fact that CENPA affects both processes at the cellular level suggests it has the potential to impact on tumor immunity.

## Discussion

In recent years, the incidence of kidney cancer has continued to rise, and its poor prognosis and high mortality rate pose a great threat and impact on patients’ lives. Therefore, the challenge is to identify precise biomarkers for prognosis assessment and targeted therapy in KIRC patients. In this study, we used the TCGA KIRC dataset to screen for genes with significant prognostic value and to identify potential prognostic indicators of KIRC. Transcription factors and m6A regulators regulate gene expression at the transcriptional and translational levels of genes (Song et al., 2020; An and Duan, 2022), respectively, regulating downstream critical genes. We found that CENPA and IGF2BP3 were most closely associated with prognosis and tumor immunity in KIRC, so we explored the function and mechanism of CENPA and IGF2BP3 in KIRC.

There are many biomarkers about m6A regulators (Zhou et al., 2019; Wan et al., 2021; Zhang et al., 2020; Chen et al., 2020; Wang et al., 2020; Zhong et al., 2021) or transcription factors (Sun et al., 2021; Zhu Y. et al., 2020) in KIRC, but few have elucidated the relationship between them. There is growing evidence that m6A is broadly involved in regulating tumorigenesis and development by influencing the expression of target genes that regulate various physiological processes, including self-renewal, invasion and proliferation. The m6A is involved in translation, degradation, splicing, export and folding, thereby affecting protein expression levels. m6A regulators target transcription factors and can influence the levels of transcription factors, providing another regulatory pathway for protein expression (Ahuja et al., 2016; Mohammad et al., 2019).

Therefore, we explored the regulatory role of IGF2BP3 on CENPA, which are found to have potential help in prognostic stratification. Given that MEF2B functions as a transcription factor influencing various processes such as cell differentiation, proliferation, and apoptosis, our bioinformatics analysis also predicts that it may be involved in the regulation of the IGF2BP3, CENPA axis. Therefore, MEF2B may also play a crucial role in regulating the IGF2BP3, CENPA axis in KIRC, thereby impacting prognosis. CENPA is a transcription factor, which plays a key role in cell cycle regulation, cell division and gene stability by ensuring the normal formation and function of centromeres and kinetochores (Shrestha et al., 2021; Giunta et al., 2021). The m6A regulators are closely related to the activation and inhibition of cancer pathways. IGF2BP3 is a post-transcriptional regulator of mRNA localization, stability and translation control (Marshall et al., 2008). IGF2BP3 is a potential oncogene spanning multiple cancer types (Bell et al., 2013; Lederer et al., 2014). We identified CENPA as a target gene for the m6A reader IGF2BP3 through the M6A2Target database, a comprehensive database of the m6A-modified writer, eraser, and reader target genes (Deng et al., 2021). Combining the results of the PPI network and correlation analysis, we inferred that IGF2BP3 may regulate CENPA protein levels, which in turn affects the transcription of downstream target genes and functions as a regulator of tumor progression.

In summary, we constructed a novel survival risk stratification model based on m6A and TF signature and elucidated the regulatory network. Their expression was verified in human tissues of KIRC patients. Our results suggest that the m6A regulator IGF2BP3 may regulate the expression of downstream genes by regulating the transcription factor CENPA, they may serve as a prognostic indicator for KIRC patients. Further studies should be conducted in the future to explore the roles and mechanisms of IGF2BP3 and CENPA. Using IGF2BP3 and CENPA as biomarkers or targets to predict patients’ tumor immune microenvironment and prognosis, as well as to serve as drug targets for combination immunotherapy, is significant for the diagnosis and treatment of KIRC, providing new possibilities for precision medicine in KIRC diagnosis and treatment.

## Data Availability

The datasets analyzed in this study are publicly available. The TCGA data for KIRCwere obtained from TCGA via the Genomic Data Commons portal: https://portal.gdc.cancer.gov/. The GEO dataset GSE40435 was downloaded from the NCBI Gene Expression Omnibus: https://www.ncbi.nlm.nih.gov/geo/query/acc.cgi?acc=GSE40435.
