# Supplementary material for: Identification of IGF2BP3 and CENPA as key regulators of immunophenotypes in renal clear cell carcinoma
Source: Front Genet. 2026 Jan 12;16:1749780. doi: 10.3389/fgene.2025.1749780 (PMC12832112; doi:10.3389/fgene.2025.1749780)
Supplement: Supplementary file 1 [file DataSheet1.docx]

Supplementary Materials

Table S1. M6A RNA modification-related genes

| Gene | Category |
| --- | --- |
| METTL3 | Writer |
| METTL14 | Writer |
| WTAP | Writer |
| VIRMA | Writer |
| RBM15 | Writer |
| RBM15B | Writer |
| ZC3H13 | Writer |
| METTL16 | Writer |
| FTO | Eraser |
| ALKBH5 | Eraser |
| YTHDC1 | Reader |
| YTHDC2 | Reader |
| YTHDF1 | Reader |
| YTHDF2 | Reader |
| YTHDF3 | Reader |
| HNRNPC | Reader |
| HNRNPA2B1 | Reader |
| IGF2BP1 | Reader |
| IGF2BP2 | Reader |
| IGF2BP3 | Reader |
| FMR1 | Reader |
| ELAVL1 | Reader |
| CBLL1 | Writer-associated |
| LRPPRC | Putative |

Table S2. M6A DEGs

| Tag | logFC | AveExpr | t | P.Value | adj.P.Val | B |
| --- | --- | --- | --- | --- | --- | --- |
| IGF2BP2 | -2.505291435 | -0.79384496 | -11.08067807 | 4.21E-26 | 5.08E-25 | 48.15482739 |
| LRPPRC | -0.622225836 | 3.994458301 | -8.368295675 | 4.07E-16 | 2.70E-15 | 25.41563089 |
| IGF2BP3 | 2.312463199 | #NAME? | 7.341744123 | 6.97E-13 | 3.70E-12 | 18.10822738 |
| FMR1 | -0.353243905 | 2.773202758 | -6.297615672 | 5.82E-10 | 2.48E-09 | 11.50134013 |
| ZC3H13 | -0.43744647 | 2.779867036 | -6.005391601 | 3.30E-09 | 1.33E-08 | 9.810658529 |
| FTO | 0.472106721 | 3.057500048 | 5.773374346 | 1.24E-08 | 4.78E-08 | 8.519123853 |
| YTHDC2 | 0.358661456 | 1.977300209 | 5.360167409 | 1.18E-07 | 4.19E-07 | 6.332393291 |
| RBM15 | 0.249080617 | 1.218680438 | 4.614136162 | 4.82E-06 | 1.46E-05 | 2.761430173 |
| METTL14 | -0.265662275 | 1.824273499 | -4.468741005 | 9.40E-06 | 2.77E-05 | 2.123229007 |
| ALKBH5 | 0.214410887 | 5.337989046 | 4.324149841 | 1.79E-05 | 5.13E-05 | 1.507539096 |
| YTHDF3 | -0.240054336 | 3.787062265 | -3.914807701 | 0.000100787 | 0.000265077 | -0.131817072 |
| WTAP | 0.190590113 | 3.787421314 | 3.677076702 | 0.00025696 | 0.000644675 | -1.012801357 |
| RBM15B | -0.186373262 | 2.749357888 | -3.602259466 | 0.000341441 | 0.000843509 | -1.279152047 |
| HNRNPA2B1 | -0.130593507 | 6.077226362 | -2.663125047 | 0.007948399 | 0.015990679 | -4.171913637 |
| YTHDF2 | -0.110363599 | 4.081333911 | -2.651374337 | 0.008226973 | 0.016496127 | -4.202764961 |
| METTL3 | 0.191826935 | 1.979713013 | 2.410761905 | 0.016217018 | 0.030778775 | -4.805124586 |
| IGF2BP1 | -0.657087922 | #NAME? | -2.25682853 | 0.024377324 | 0.044539 | -5.160714661 |
| HNRNPC | -0.091221592 | 5.482249374 | -2.188038862 | 0.029049396 | 0.052153209 | -5.312598983 |
| VIRMA | 0.082019337 | 2.466474431 | 1.444470029 | 0.149125636 | 0.219307683 | -6.654949747 |
| YTHDC1 | -0.047338 | 3.540366349 | -0.84230091 | 0.399953123 | 0.487865766 | -7.341611471 |
| CBLL1 | 0.030414184 | 2.374355497 | 0.493543253 | 0.621808295 | 0.686919226 | -7.574283579 |
| YTHDF1 | 0.012392789 | 3.957318317 | 0.327398099 | 0.743480309 | 0.790229707 | -7.64243604 |
| ELAVL1 | 0.008412822 | 3.306231124 | 0.264131357 | 0.79176885 | 0.831288422 | -7.661138392 |
| METTL16 | -0.003381136 | 1.968253185 | -0.056516993 | 0.95494866 | 0.964179219 | -7.694409576 |


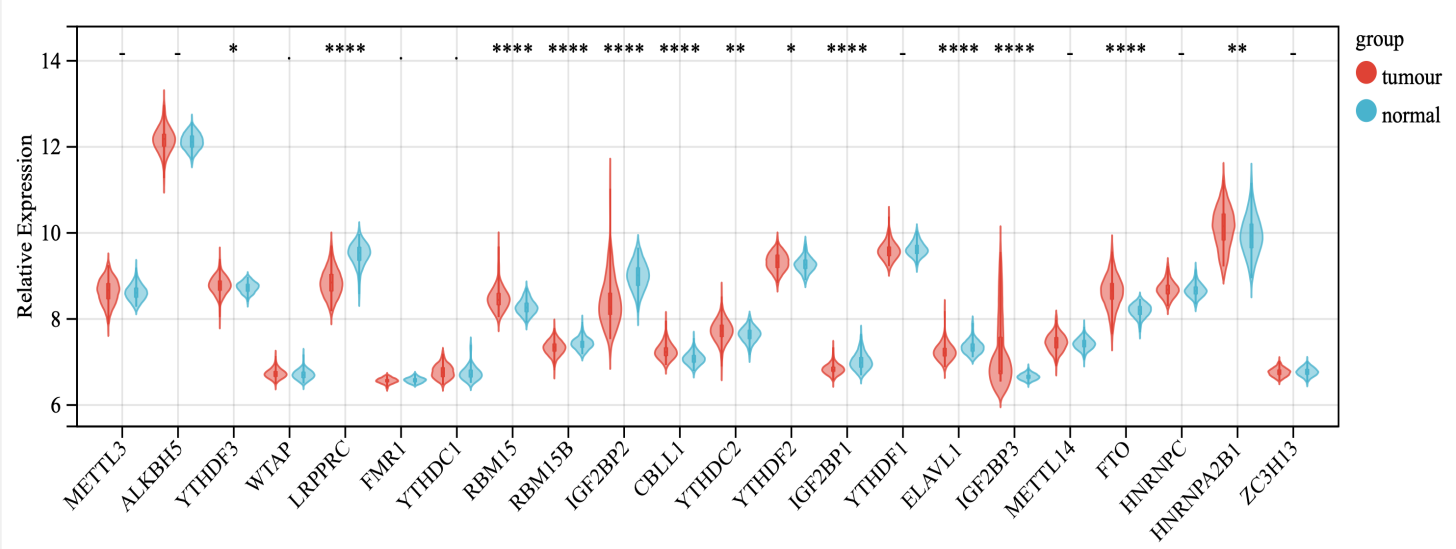


Figure S1. Expression of M6A RNA modification-related genes' mRNA in the GEO dataset GSE40435.


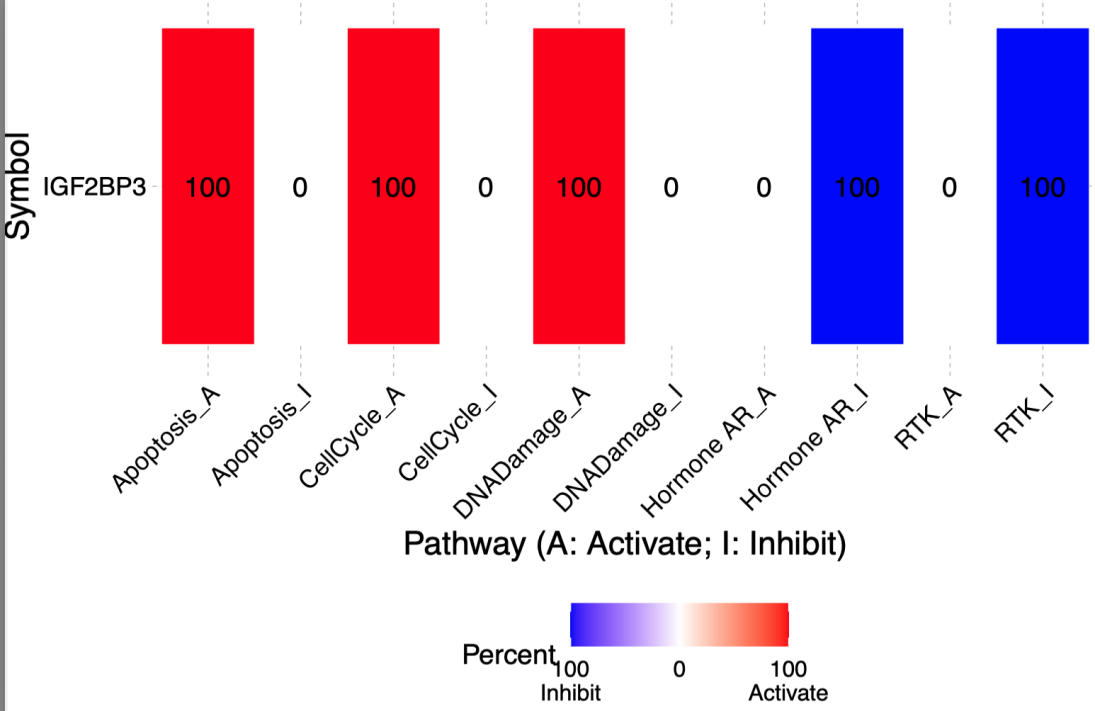


Figure S2. The IGF2BP3 gene's mRNA expression has potential effect on pathway activity.


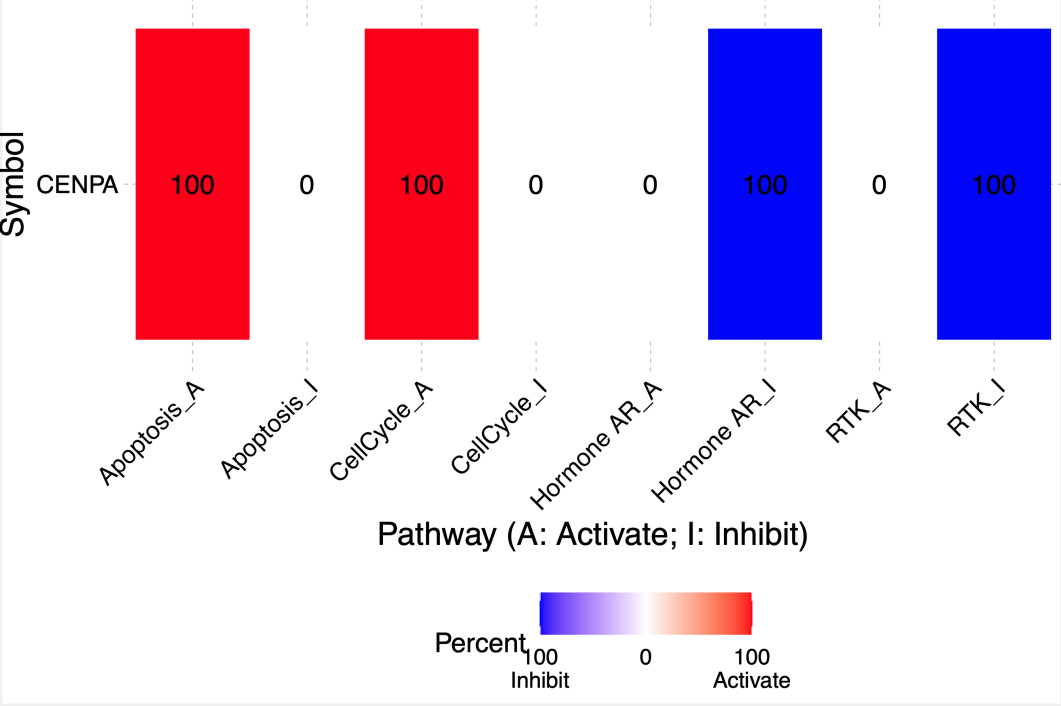


Figure S3. The CENPA gene's mRNA expression has potential effect on pathway activity.
